# Supportive Care in Hemato-Oncology: A Review in Light of the Latest Guidelines

**DOI:** 10.5505/tjh.2012.10327

**Published:** 2012-03-05

**Authors:** Eren Gündüz, Zafer Gülbaş

**Affiliations:** 1 Eskişehir Osmangazi University, School of Medicine, Department of Hematology, Eskişehir, Turkey; 2 Anadolu Health Center, Bone Marrow Transplantation Center, Kocaeli, Turkey

**Keywords:** Hematology, Supportive care, Nausea/vomiting, Anemia, Neutropenia

## Abstract

Recent developments in cancer therapy have resulted in increases in treatment success rates and survival. One of thebasic goals of such therapy is improving patient quality of life. Chemotherapy protocols for solid or hematologicalmalignancies-most of which include multiple agents-negatively impact patient quality of life. Additionally, there havebeen developments in supportive care, which seeks to ameliorate or minimize the negative effects of chemotherapy.Herein we present a review and brief summarization of some of the agents used for supportive care in cancer patientsin light of the latest guidelines.

## Abstract (tr)

Son yıllarda kanser tedavisi alanında sağlanan gelişmeler hastaların tedavi şanslarının artması ve yaşam sürelerinde uzama ile sonuçlanmıştır. Bu sıkıntılı tedavi sürecinde yaşam kalitesinin arttırılması temel hedeflerden biri olmalıdır. Solid ya da hematolojik maligniteler için verilen çoğu çoklu ajanlar içeren kemoterapi protokolleri hastaların yaşam kalitesini olumsuz etkiler. Bu olumsuz etkilerden hastayı kurtarmak ya da en az hasar görmesi sağlamak amacıyla yapılan destek tedavilerde de gelişmeler vardır. Bu derlemede, destek tedavi olarak verilen bu ajanlardan bazılarını en son kılavuzlar ışığında kısaca özetledik.

## INTRODUCTION

Supportive care aims to ameliorate the adverse effectsof chemotherapy, and to prevent reductions in the chemotherapydose and delays in its schedule. These adverseeffects include nausea/vomiting, diarrhea, constipation,pain, infections, cytopenia, allergic reactions, mucositis,osteoporosis, and neuropathy. Cancer patient quality oflife increases with supportive care. The success of treatmentincreases along with the level of treatment compliance.Supportive care is critical in intolerant and elderlypatients with multiple comorbidities. Chemotherapy and/or radiotherapy target the disease, whereas patient quality of life is the target of supportive care. Physicians sometimesoverlook developments in supportive care, as theyprimarily concentrate on disease-targeted therapy. Hereinwe present a review of supportive care in light of the latestguidelines, focusing only on nausea/vomiting, anemia,and myeloid growth factors, as each side effect of cancertreatment warrants individual attention.

**Chemotherapy-Induced Nausea/Vomiting**

Chemotherapy-induced nausea/vomiting (CINV ) is acommon adverse event associated with cancer treatmentthat occurs in 70%-80% of patients undergoing chemotherapy.CINV results in significant morbidity and negativelyaffects quality of life [1,2]. The risk of CINV is associatedwith the type of chemotherapy, and increases withage <50 years, female gender, a history of CNIV duringchemotherapy, pregnancy-induced nausea/vomiting, ahistory of motion sickness, and anxiety [3,4]. Chemotherapeuticagents cause vomiting via activation of neurotransmitterreceptors located in the chemoreceptor trigger zone,gastrointestinal tract, and vomiting center. Serotonin, substanceP, and dopamine receptors are the primary neuroreceptorsinvolved in the emetic response [5].

CINV is classified into 5 categories: acute, delayed,anticipatory, breakthrough, and refractory. Acute-onsetCINV refers to nausea and/or vomiting that occurs within24 h of chemotherapy administration [3]. Nausea and/orvomiting that develop >24 h after chemotherapy administrationis known as delayed emesis [2]. Anticipatory nauseaand/or vomiting occur prior to the administration ofnext chemotherapy; because it is a conditioned response,it can occur only after a negative past experience withchemotherapy [6]. Vomiting that occurs within 5 d ofprophylactic antiemetic use or requires rescue antiemetictreatment is known as breakthrough emesis. Vomiting inresponse to subsequent chemotherapy cycles that followfailed prophylactic and/or rescue antiemetic treatmentduring previous cycles is known as refractory emesis [7].

**Antiemetic Agents**

**1. Dopamine Receptor Antagonists**

Dopamine receptors are located in the chemoreceptortrigger zone and dopamine receptor antagonists primarilyaffect this area; however, high doses of dopamine receptorblockades result in extrapyramidal reactions, disorientation,and sedation, which limit the clinical use of suchagents, including phenothiazines and butyrophenones(droperidol and haloperidol) [8].

**2. Serotonin (5-HT3) Receptor Antagonists**

Serotonin receptors—specifically 5-HT3 receptors—arepresent in the central nervous system and gastrointestinaltract. First-generation 5-HT3 receptor antagonists (azasetron,dolasetron, granisetron, ondansetron, ramosetron,and tropisetron) are equally effective and toxic when usedat the recommended doses, and differ only in terms of cost.The primary symptoms of their toxicity are mild headache,constipation, and occasional diarrhea. The second-generation5-HT3 receptor antagonist palonosetron might moreeffectively control delayed CINV than the first-generation5-HT3 receptor antagonists [8]

**3. Dopamine-serotonin Receptor Antagonists**

Metoclopramide has antiemetic properties, both at lowdoses as a dopamine antagonist and at high doses as aserotonin antagonist. Use of a relatively high dose (20 mgt.i.d. p.o.) may result in sedation and extrapyramidal sideeffects [9,10].

**4. Substance P (Neurokinin-1) ReceptorAntagonists**

Substance P is a mammalian tachykinin in the vagalafferent neurons that innervate the brainstem nucleus tractussolitarius, which sends impulses to the vomiting center.Substance P induces vomiting and binds to neurokinin1 (NK-1) receptors in the abdominal vagus, the nucleustractus solitarius, and the area postrema. Compounds thatblock NK-1 receptors, including vofopitant, CP-122,721,CJ-11,794, fosaprepitant (L758,298), aprepitant (MK-869), and casopitant, reduce emesis following cisplatin,ipecac, apomorphine, and radiation therapy [8,11].

**5. Corticosteroids**

Corticosteroids have been shown to be effective in theprevention of CINV , although their antiemetic mechanismof action remains unknown. The control of CINV is markedlyenhanced when corticosteroids are used in combinationwith 5-HT3 and NK-1 receptor antagonists [12,13].The most widely used corticosteroid antiemetic is dexamethasone[8].

**6. Olanzapine**

Olanzapine is an antipsychotic that blocks multipleneurotransmitters, including dopamine at the D1, D2, D3,and D4 brain receptors, serotonin at the 5-HT2a, 5-HT2c,5-HT3, and 5-HT6 receptors, catecholamines at alpha 1adrenergic receptors, acetylcholine at muscarinic receptors,and histamine at H1 receptors [14,15]. Common sideeffects are sedation, weight gain, and an association withthe onset of diabetes mellitus [16,17,18]. Olanzapine’s antiemetic property is due to its activity at multiple receptorsinvolved in nausea and emesis [8].

**7. Gabapentin**

The anticonvulsant gabapentin has been reportedto reduce delayed nausea in a small number of patientsundergoing adjuvant chemotherapy for breast cancer;however, additional research is necessary to determine itsefficacy more precisely [19].

**8. Cannabinoids**

Cannabinoid receptors of the CB1 type are present inthe area postrema, nucleus tractus solitarius, and dorsalmotor nucleus, which are key sites of emetogenic controlin the brainstem. Cannabinoid CB2 receptors are presenton brainstem neurons and may play a role in mediatingthe effects on emesis [20,21]. Dronabinol and nabilonehave been approved by the US FDA for use in CINVrefractory to conventional antiemetic therapy, but the roleof cannabinoids in the prevention of CINV remains to beestablished [22].

**Clinical Management of CINV**

All of the following recommendations are those of theNational Comprehensive Cancer Network (NCCN) PracticeGuidelines in Oncology v.2.2010 [23].

**1. Emesis Prevention For High Emetic RiskIntravenous Chemotherapy**

Data for post-cisplatin (≥50 mg m–2) emesis preventioncategory 1; others are category 2A.

**Serotonin (5-HT3) antagonist**

Dolasetron 100 mg p.o. or 1.8 mg kg–1 IV on d 1

or

Granisetron 2 mg p.o., 1 mg b.i.d. p.o., 

or 

0.01 mg kg–1(maximum: 1 mg) IV on d 1orOndansetron 16-24 mg p.o. or 8-12 mg (maximum: 32mg d–1) IV on d 1

or

Palonosetron 0.25 mg IV on d 1 

and

**Steroid**

Dexamethasone 12 mg p.o. or IV on d 1 and 8 mg d–1 p.o.on d 2-4 

and

**Neurokinin 1 antagonist**

Aprepitant 125 mg p.o. on d 1 and 80 mg d–1 p.o. on d 2-3

or

Fosaprepitant 115 mg IV on d 1 only, and then aprepitant80 mg d–1 p.o. on d 2-3± Lorazepam 0.5-2 mg p.o. or IV± H2 blocker or proton pump inhibitor

**2. Emesis Prevention for Moderate Emetic Risk Intravenous Chemotherapy**

**Day 1**

**Serotonin (5-HT3) antagonist**

Dolasetron 100 mg p.o., 1.8 mg kg–1 IV, or 100 mg IV(category 1)

or

Granisetron 1-2 mg p.o., 1 mg b.i.d. p.o. (category 1), 

or

0.01 mg kg–1 (maximum: 1 mg) IV,

or

Ondansetron 16-24 mg p.o. 

or

8-12 mg (maximum: 32mg d–1) IV (category 1)

or

Palonosetron 0.25 mg IV on d 1 only

and

**Steroid**

Dexamethasone 12 mg p.o. or IV

**with/without**

**Neurokinin 1 antagonist**

Aprepitant 125 mg p.o.

Fosaprepitant 115 mg IV on d 1 only

± Lorazepam 0.5-2 mg p.o. or IV

± H2 blocker or proton pump inhibitor

**Day 2-3**

**Serotonin (5-HT3) antagonist monotherapy**

Dolasetron 100 mg d–1 p.o. , 1.8 mg kg–1 IV, or 100 mg IV,

or

Granisetron 1-2 mg d–1 p.o., 1 mg b.i.d. p.o., 

or

0.01 mgkg–1 (maximum: 1 mg) IVor 

Ondansetron 8 mg b.i.d. p.o., 16 mg d–1 p.o., 

or

8 mg(maximum: 32 mg d–1) IVor

**Steroid monotherapy**

Dexamethasone 8 mg d–1 p.o. or IV

or

**Neurokinin 1 antagonist ± steroid**

Aprepitant 80 mg p.o. ± dexamethasone 8 mg d–1 p.o. or IV

± Lorazepam 0.5-2 mg p.o. or IV

± H2 blocker or proton pump inhibitor

**3. Emesis Prevention for Low and Minimal EmeticRisk Intravenous Chemotherapy**

No routine prophylaxis is recommended for minimalemetic risk intravenous chemotherapy.

Dexamethasone 12 mg d–1 p.o. or IV

or

Metoclopramide 10-40 mg or IV, and then every 4 or 6 h

or

Prochlorperazine 10 mg p.o. or IV, and then every 4 or 6 h

± Lorazepam 0.5-2 mg p.o. or IV every 4 or 6 h

± H2 blocker or proton pump inhibitor

**4. Breakthrough Treatment for CINV**

The general principle is to add 1 agent of a different classto the current regimen.

**Antipsychotic**

Haloperidol 1-2 mg p.o. every 4-6 h

Olanzapine 2.5-5 mg b.i.d. p.o. (category 2B)

**Benzodiazepine**

Lorazepam 0.5-2 mg p.o. every 4 or 6 h

**Cannabinoid**

Dronabinol 5-10 mg every 3 or 6 hNabilone 1-2 mg b.i.d. p.o.

**Dopamine receptor antagonist**

Metoclopramide 10-40 mg p.o. or IV every 4 or 6 h

**Phenothiazine**

Prochlorperazine 10 mg p.o. or IV every 4 or 6 h

Promethazine 12.5-25 mg p.o. or IV every 4 h

**Serotonin (5-HT3) antagonist**

Dolasetron 100 mg d–1 p.o., 1.8 mg kg–1 IV, or 100 mg IV

Granisetron 1-2 mg d–1 p.o., 1 mg b.i.d. p.o., or 0.01 mgkg–1 (maximum: 1 mg) IV

Ondansetron 16 mg d–1 p.o. or 8 mg d–1 IV

**Steroid**

Dexamethasone 12 mg d–1 p.o. or IV

**5. Anticipatory Emesis Prevention/Treatment**

Alprazolam 0.5-2 mg t.i.d. p.o. beginning the night before treatment 

or

Lorazepam 0.5-2 mg p.o. on the night before and morningof treatment

**Cancer and Chemotherapy-Induced Anemia**

Anemia is a frequent complication of cancer and occursin 30%-90% of patients [24]. At the time of diagnosis30%-40% of patients with non-Hodgkin’s lymphoma orHodgkin’s lymphoma, and ≤70% of patients with multiplemyeloma are anemic; rates are higher among patients withmyelodysplastic syndromes. Among patients with solidcancers or lymphomas, ≤50% develop anemia followingchemotherapy [25]. Anemia is a frequent cause of morbidityand might increase mortality [26].

Tumor cells activate the immune system of the host anda number of cytokines are produced. This inflammatoryresponse affects erythropoietin production, suppressesburst-forming unit-erythroid, and colony-forming uniterythroid,and impairs iron utilization. Tumor cells mayalso decrease erythrocyte survival either via tumor necrosisfactor or by causing erythrophagocytosis [27]. Nutritionaldeficiency, hemolysis, bleeding, hereditary diseases,renal insufficiency, and anemia of chronic disease canalso contribute to anemia in cancer patients [28,29]. Themyelosuppressive effects of chemotherapy and radiationtherapy are also significant factors associated with anemia[30,31]. Anemia can be corrected by treating the underlyingetiology, transfusion with packed red blood cells, orerythropoiesis stimulating agents, with or without ironsupplementation.

The NCCN concurs that a hemoglobin level ≤11 g dL–1in cancer patients should be investigated. In patients witha high baseline level, a drop of ≥ 2g dL–1 should also beassessed. There are 3 general anemia categories describedby the NCCN:

1. Asymptomatic anemia without significant comorbidity,for which observation and periodic reevaluation areappropriate;

2. Asymptomatic anemia with comorbidity or high risk,for which transfusion should be a consideration;

3. Symptomatic anemia, for which transfusion should beperformed.

If the hemoglobin level decreases following chemotherapy,transfusion may be appropriate even in the absenceof symptoms or significant comorbidity [23]. Packed redblood cell (PRBC) transfusion is the only treatment optionin patients that require immediate correction of anemia.Risks associated with PRBC transfusion include transfusion-related reactions, congestive heart failure, bacterialcontamination, viral infections, iron overload, and anincrease in thrombotic events [32].

Administration of erythropoiesis-stimulating agents(ESAs) decrease the need for PRBC transfusion in cancerpatients undergoing chemotherapy [33-35]; however,there are risks associated with ESA therapy, including anincrease in mortality, and an increase in tumor progressionof breast cancer [36], head and neck cancer [37],cervical cancer [38], non-small cell lung cancer [39], nonmyeloidcancer [40], and lymphoid malignancy [41]. Elevatedthromboembolic risk has also been associated withESA treatment [42-44]. Hypertension/seizures and purered cell aplasia 90% of occured with epoetin alfa have alsobeen reported in chronic renal failure [23]. In addition tosafety concerns, ESAs also have considerable impact onhealthcare financial resources [45].

Historically, ESA treatment strategies were designedto achieve and maintain hemoglobin levels >12 g dL–1,decrease the need for transfusion, and improve patientquality of life [46]. In 2008 the US FDA prohibited useof ESAs in cancer patients seeking cure. Reimbursementis limited to patients with hemoglobin levels <10 g dL–1[25]. The University of Texas MD Anderson Cancer Centermandates that following initial administration of ESAs,subsequent doses be given only to those with a hemoglobinlevel <11 g dL–1, leading to intermittent treatment versusthe once standard continuous treatment pattern [47].Myelodysplastic syndrome patients with low intermediate-1 IPSS risk, hemoglobin <10 g dL–1, and serum erythropoietin<500 mIU mL–1 should be considered for ESAtreatment [48].

According to the package insert dosing schedule, theinitial dose of epoetin alfa is 150 U kg–1 t.i.w; the dose canbe increased to 300 U kg–1 t.i.w. if there is no responseafter 4 weeks. The initial dose of epoietin beta is 30,000 IUweek–1 and the dose can be increased to 60,000 IU week–1in there is no response after 4 weeks. The initial dose ofdarbepoetin alfa is 2.25 μg kg–1 QWK; the dose can beincreased to 4.5 μg kg–1 QWK if there is no response. Thedose should be adjusted individually for each patient, soas to maintain the lowest hemoglobin level sufficient toavoid red blood cell transfusion. If the hemoglobin level issuch that transfusion is unnecessary or increases >1 g dL–1in any 2 week period the epoetin alfa or epoetin beta doseshould be reduced by 25%, and the darbepoetin alfa doseshould be reduced by 40%.

If ferritin is ≤800 ng mL–1 and transferrin saturationis <20%, IV iron supplementation should be consideredalong with erythropoietin therapy; however, patients withactive infection should not receive IV iron therapy. IV Irondextran 100 mg is administered over the course of 5 minQWK for 10 doses or as a 1-g infusion administered duringthe course of several hours. Ferric gluconate is administeredas 125 mg IV over the course of 60 min QWK for8 doses or as 200 mg IV over the course of 3-4 h repeatedevery 3 weeks for 5 doses. Iron sucrose is given as 200 mgIV over the course of 60 min every 2-3 weeks or as 200 mgIV over the course of 2-5 min every 1-4 weeks [23].

**Myeloid Growth Factors**

Myelosuppression is the major dose-limiting toxicityassociated with many chemotherapy regimens and can alsoresult in chemotherapy schedule delay, compromising theeffectiveness of chemotherapy [49-52]. Infections associatedwith neutropenia may be accompanied by sepsis andoccasionally death. Severe myelosuppression is accompaniedby impaired quality of life, even in the absence offever [53]. Myeloid growth factors stimulate proliferationof neutrophil progenitors and enhance neutrophil function.The use of myeloid growth factors is designed toreduce the duration of myelosuppression and the depth ofneutropenia, and decrease the likelihood of infection [54].

A meta-analysis of myeloid growth factors trialsreported that there were significant reductions in severeneutropenia, neutropenic fever, and infections in patientstreated for non-Hodgkin’s lymphoma and Hodgkin’s lymphoma[55]. Trials of myeloid growth factors in patientstreated for acute leukemia indicate they can reduce theduration of both neutropenia and hospitalization duringinduction therapy; however, their benefit is modest, andremission and survival rates associated with their use areinconsistent. The concern that using myeloid growth factors may interfere with the evaluation of remission maybe dealt with delaying the start of growth factors untilafter the day 14 bone marrow and stopping at neutrophilrecovery several days prior to performing the bone marrowbiopsy to assess remission. Stimulation of leukemiccell proliferation has not been observed in clinical trials.Recruitment leukemia into cycling, making the leukemiacells more sensitive to chemotherapy, has also not demonstratedconvincing evidence of clinical benefit. Thus, use ofgranulocyte colony-stimulating factor (G-CSF) in patientswith acute leukemia should be based only on preventingneutropenic complications. During post-remission consolidationtherapy the benefits may be more substantial[54,56].

The most common toxicity associated with G-CSFtherapy is mild-to-moderate bone pain, which is usuallyeffectively controlled with non-narcotic analgesics. Therehave also been reports of splenic rupture in patients treatedwith G-CSF [54]. A retrospective review reported that ahigh rate of bleomycin toxicity has been linked to G-CSFuse in Hodgkin’s lymphoma patients receiving bleomycincontainingtherapy [57]. Some patients develop allergicskin, respiratory system, and cardiovascular system reactions[58].

Primary prophylaxis is achieved via administration ofmyeloid growth factors during the initial chemotherapycycle, in anticipation of the risk of neutropenic complications.The use of prophylactic myeloid growth factorsis recommended for solid tumor/lymphoma patients thathave ≥20% likelihood of developing fever; in patients witha 10%-20% risk of fever G-CSF should be considered ifthere are additional risk factors (advanced age, history ofchemotherapy or radiotherapy, and pre-existing neutropenia,or tumor involvement in the bone marrow, poorperformance status, and comorbidity, including renal andliver dysfunction). G-CSF should not be routinely used inpatients with a <10% risk of fever. According to AmericanSociety of Clinical Oncology (ASCO) guidelines, secondaryprophylaxis with G-CSF should be considered if maintainingthe dose intensity is considered to be important[59-62].

Compared to its prophylactic use, there is less evidencesupporting the therapeutic use of G-CSF for febrileneutropenia as an adjunct to antibiotics [63-65]. Patientswith febrile neutropenia given prophylactic filgrastim orsargramostim should continue with G-CSF therapy; however,as pegfilgrastim is long acting patients given prophylacticpegfilgrastim should not be treated with additionalG-CSF [66]. Currently, there is a lack of evidence supporting the therapeutic use of pegfilgrastim; therefore,only filgrastim or sargramostim should be administered inthe therapeutic setting. In patients that have not receivedprophylactic G-CSF the NCCN recommends evaluatingthe risk factors for infection-related complications or poorclinical outcome, including advanced age (>65 years),sepsis syndrome, severe (absolute neutrophil count <100μL) or anticipated prolonged (>10 d) neutropenia, pneumonia,invasive fungal infection or other clinically documentedinfections, hospitalization, and a history of febrileneutropenia. If risk factors are present G-CSF should beconsidered.

Myeloid growth factors currently used for the prophylaxisof febrile neutropenia and maintenance of scheduleddose delivery include filgrastim, pegfilgrastim (category1), and sargramostim (category 2B). Filgrastim treatmentis initiated within 1-3 d after the completion of chemotherapyat a dose of 5 μg·kg–1·d–1 until post nadir absoluteneutrophil count (ANC) recovery is normal or nearnormal, according to laboratory standards. The dose maybe rounded to the nearest vial site by intitution definedweight limits. Moreover, evidence exists that supports theinitiation of pegfilgrastim 24 h after completion of chemotherapy,administered every 3 weeks at a dose of 6 mgfor each chemotherapy cycle. Same-day administration offilgrastim or pegfilgrastim (within 24 h of the completionof chemotherapy) is not recommended [67,68]

## CONCLUSION

By means of all summarized supportive care interventionswe are able to better treat our patients, prolong their survival and decrease complications of cancer chemotherapy.New therapies may add new complications butsupportive care is also improving. If we know the complicationsof our therapy we can be able to choose the suitable supportive care intervention to increase the quality of life. Supportive care must be a more essential part of main therapy in the future.

## CONFLICT OF INTEREST STATEMENT

The authors of this paper have no conflicts of interest, including specific financial interests, relationships, and/ or affiliations relevant to the subject matter or materials included.
